# Highly Efficient Perovskite Solar Cell Based on PVK Hole Transport Layer

**DOI:** 10.3390/polym14112249

**Published:** 2022-05-31

**Authors:** Yao Xu, Qiaoli Niu, Ling Zhang, Chaochao Yuan, Yuhui Ma, Wei Hua, Wenjin Zeng, Yonggang Min, Jingsong Huang, Ruidong Xia

**Affiliations:** 1 Key Laboratory for Organic Electronics and Information Displays, Institute of Advanced Materials, Jiangsu National Synergetic Innovation Center for Advanced Materials (SICAM), Nanjing University of Posts and Telecommunications, 9 Wenyuan Road, Nanjing 210023, China; xy18936032151@163.com (Y.X.); zl13657132936@163.com (L.Z.); ycc161217@163.com (C.Y.); 1016061506@njupt.edu.cn (Y.M.); huawei00630@163.com (W.H.); iamwjzeng@njupt.edu.cn (W.Z.); 2 The School of Materials and Energy, Guangdong University of Technology, Guangzhou 510006, China; iamygmin@njupt.edu.cn; 3 Oxford Suzhou Centre for Advanced Research (OSCAR), University of Oxford, 388 Ruoshui Road, Suzhou 215000, China; jingsong.huang@oxford-oscar.cn

**Keywords:** PVK, NPB, hole transport layer, perovskite solar cell, small molecule doping

## Abstract

A π-conjugated small molecule N, N’-bis(naphthalen-1-yl)-N, N’-bis(phenyl)benzidine (NPB) was introduced into poly(9-vinylcarbazole) (PVK) as a hole transport layer (HTL) in inverted perovskite solar cells (PSCs). The NPB doping induces a better perovskite crystal growth, resulting in perovskite with a larger grain size and less defect density. Thus, the V_OC_, J_SC,_ and FF of the PSC were all enhanced. Experimental results show that it can be ascribed to the reduction of surface roughness and improved hydrophilicity of the HTL. The effect of NPB on the aggregation of PVK was also discussed. This work demonstrates the great potential of PVK as the HTL of PSCs and provides an attractive alternative for HTL to realize high-efficiency PSCs.

## 1. Introduction

In recent past decades, organic metal halide perovskite solar cells (PSCs) have attracted tremendous attentions due to their excellent power conversion efficiency (PCE), low cost, and low temperature solution processing technology [1,2,3]. Remarkably, the PCE of PSCs has rapidly increased from 3.8% in 2009 to 25.5% [4,5]. The high PCE of PSCs is mainly due to the outstanding optoelectronic properties of perovskite, such as excellent light absorption coefficient, long electron–hole diffusion length, and high defect tolerance [6].

In p-i-n-type PSCs, the perovskite layer is sandwiched between a p-type hole transporting layer (HTL) and an n-type electron transporting layer (ETL). Therefore, besides the componential design and morphological optimization of the perovskite layer, the charge transport materials have been found to significantly influence the device performance [7,8]. Inorganic oxide, organic small molecules, and polymers are all commonly used in hole transport materials (HTMs). Polymer HTM has better film-forming properties and a higher compatibility with low-cost solution processing technology [9,10,11]. Poly (3,4-ethylenedioxythiophene): poly (4-styrenesulfonate) (PEDOT: PSS) was the first polymer to be used as an HTL due to its scale-up processing properties, while PSCs based on PEDOT:PSS usually suffered from low open circuit voltage (V_OC_) due to the inferior band gap alignment and the serious interface recombination [12,13]. Poly-bis (4-phenyl) (2,4,6-trimethylphenyl) amine (PTAA) was then used to substitute PEDOT: PSS, based on which the V_OC_ of the PSC reached above 1 eV. However, PTAA has a strong hydrophobic property, which makes the wet chemical deposition of perovskite a great challenge [14]. Thus, it is urgent to find better options for HTL. Poly(9-vinylcarbazole) (PVK) is also a p-type semiconducting polymer, which has been widely used as the HTL in organic electronic devices [15,16]. It can be easily deposited by solution casting technology at room temperature [17]. In terms of PSCs based on PVK HTL, the N atom in PVK can form hydrogen bonds with I^−^ ions in perovskite and interact with Pb^2+^ ions in perovskite to passivate defects in the perovskite layer [18,19]. This promises to make PVK a potential attractive alternative for the HTL of PSCs. However, the drawbacks of PVK HTL, such as energy level mismatch with the perovskite layer, rough surface, and relatively low hole transport mobility, seriously hinder the acquisition of highly-efficient PSCs [20].

In this study, N, N’-bis(naphthalen-1-yl)-N, N’-bis(phenyl)benzidine (NPB) was incorporated into PVK to improve the performance of PVK HTL-based PSCs. Experimental results show that because of the improvement of surface roughness and hydrophilicity of PVK HTL by doping NPB, the MAPbI_3_ film with a larger grain size and less defect density was obtained. Meanwhile, the energy gap between the highest occupied molecular orbital (HOMO) of HTL and MAPbI_3_ was reduced. Eventually, the V_OC_, J_SC,_ and FF values of the PSCs were all enhanced, resulting in a PCE promotion from 11.23% to 15.04%. The working mechanism of NPB in PVK was analyzed. This work demonstrated the great potential of PVK as the HTL of PSCs, which is an attractive alternative for the HTL of PSCs.

## 2. Materials and Methods

### 2.1. Materials

PVK, PbI_2_ (99.99%), MAI, [6,6]-phenyl C61 butyric acid methyl ester (PCBM) (99.5%), 2,9-dimethyl-4,7-diphenyl-1,10-phenanthroline (BCP), DMF (99.8%), and chlorobenzene (CB) were all purchased from Sigma-Aldrich, St. Louis, MO, USA. NPB, Pb(Ac)_2,_ and indium tin oxide (ITO, 7 Ω, sq^−1^) were purchased from Xi’an polymer light technology corp, Xi’an, China. All materials were used as received without further purification.

Perovskite (MAPbI_3_) precursor solution was prepared by mixing the MAI powder, PbI_2,_ and Pb (Ac)_2_ at a mole ratio of 2.2: 0.4: 0.6 in DMF under magnetic stirring for 6 h. PVK, NPB, and PCBM were separately dissolved in CB with concentrations of 1 mg mL^−1^, 5 mg mL^−1^, and 20 mg mL^−1^, respectively.

### 2.2. Device Fabrication

PSCs were fabricated with device structures of ITO/PVK/MAPbI_3_ (260 nm)/ PCBM (60 nm)/BCP (10 nm)/Ag (100 nm). The thickness of PVK was too thin to be detected. Before use, ITO glass substrate was cleaned with detergent, deionized water, acetone, and anhydrous ethanol in an ultrasonic bath for 20 min each. After being dried at 80 °C for 30 min, the ITO substrate underwent a O_3_ plasma treatment for 3 min to remove any organic residues. As a result, PVK layer should be deposited. The thickness of PVK layer was optimized by changing the concentration of PVK solution from 0.5 mg/mL and 1 mg/mL to 1.5 mg/mL and the spin coating speed from 2000 rpm and 4000 rpm to 6000 rpm. The J–V curves of PSCs based on PVK layer deposited with different concentrations and spin-coating speeds are shown in Figure S1, and the corresponding performance parameters are summarized in Table S1. It demonstrates that the device efficiency is the best when the solution concentration is 1 mg/mL and the spin coating speed is 4000 rpm. Therefore, 1 mg/mL PVK solution was spin-coated on ITO at 4000 rpm for 30 s in a N_2_-filled glovebox and then annealed at 160 °C for 15 min. Next, perovskite precursor solution was spin-coated at 4000 rpm for 30 s and then heated on a hot plate at 100 °C for 20 min. Then, PCBM solution was spin-coated at 1200 rpm for 30 s and then heated on a hot plate at 100 °C for 3 min. Finally, 10 nm-thick BCP and 100 nm-thick Ag were evaporated sequentially as the interface layer and top metal electrode, respectively, under a pressure of 9 × 10^−5^ Pa. The active area was 0.096 cm^2^.

### 2.3. Measurements and Characterizations

Current density–voltage (J–V) curves of the devices were measured by a Keithley 2400 Source Meter under an illumination of 1 sun (100 mW cm^−2^ AM 1.5 G, generated by a solar simulator Oriel Sol3A, Newport Corp., Irvine, CA, USA). It was calibrated with a standard Si photodiode. The step size of voltage scan was 0.01715 V. External quantum efficiency (EQE) measurements were carried out with QE-R3011 (Enlitech, Kaohsiung, Taiwan, China). The morphology of perovskite films was detected by field emission scanning electron microscopy (FESEM, S4800 microscope, Hitachi Ltd., Tokyo, Japan). Cross-sectional SEM image of PSC was tested by using field emission scanning electron microscopy (FEI, Apreo, Thermo Fisher Scientific, USA). Ultraviolet photoelectron spectroscopy (UPS) was studied using a PHI Quantera SXM (ULVAC-PHI Inc., Tokyo, Japan). The electrochemical impedance spectrum (EIS) was tested using an electrochemical workstation (Zahner, Germany) under dark conditions. Atomic force microscope (AFM) was used to investigate the surface morphology of HTL (Bruker Dimension^®^ Icon™, Bruker Corporation, Germany). X-ray diffraction (XRD) patterns of the films were collected by a Bruker D8 ADVANCE X-ray diffractometer (Bruker Corporation, Germany) under the operation conditions of 40 kV and 40 mA. Ultraviolet–visible (UV–vis) absorption measurements were carried out on a Lamba 35 spectrophotometer (Perkin-Elmer, Waltham, MA, USA). PL spectra were obtained by using FLSP920 spectrometer (Edinburgh Instruments Ltd. Livingston, UK). The contact angles were recorded on a DSA20 contact angle measurement (KRUSS, Hamburg, Germany). All the above measurements were carried out in atmosphere, and the devices were not encapsulated.

## 3. Results

### 3.1. Device Performance 

**Figure 1:**
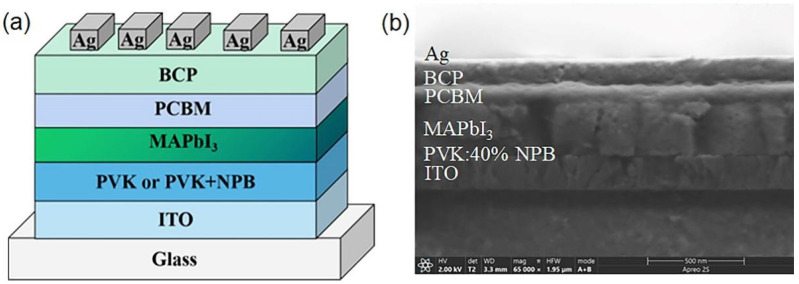
The schematic diagram of device structure (**a**) and cross-sectional SEM image (**b**) of PSC.

To examine the effect of NPB doping on the performance of PSCs, devices with a structure of ITO/HTL/MAPbI_3_ (260 nm)/PCBM (60 nm)/BCP (10 nm)/Ag (100 nm) were fabricated. PVK and PVK doped with 20 wt%, 40 wt%, and 60 wt% NPB were used as the HTL of the PSC, respectively. The schematic diagram and cross-sectional SEM image of the PSC are shown in Figure 1. 

**Figure 2:**
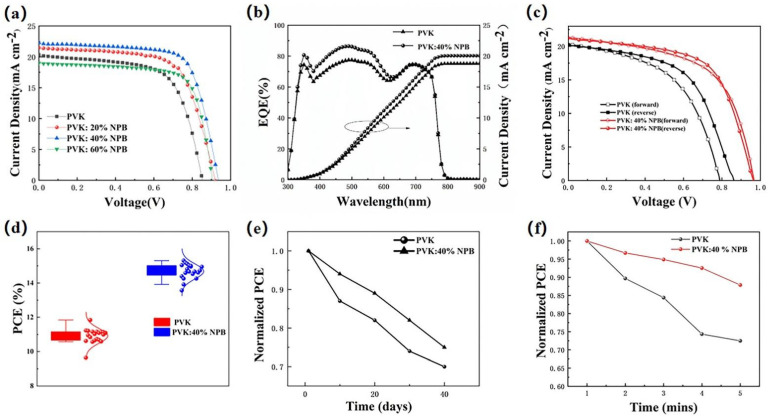
(**a**) J–V curves of PSCs; (**b**) external quantum efficiency and integrated J_SC_ of PSCs; (**c**) the reverse and forward scanned J–V curves of control device and device based on PVK: 40% NPB HTL; (**d**) PCE statistics of PSCs; (**e**) normalized PCE values during storage in glovebox filled with N_2_; and (**f**) stability of PSCs under continuous illustration by 1 sun (100 mW cm^−2^ AM 1.5 G).

The current density–voltage (J–V) curves of PSCs are shown in Figure 2a, and the detailed performance parameters are summarized in Table 1. The results show that the PCE of the control device was 11.23% with a V_OC_ of 0.85 V, a J_SC_ of 20.23 mA cm^−2^, and an FF of 65.11%. After doping NPB in PVK, the PCE values of the PSCs all increased, which shows a dependence on the NPB concentration. When the weight ratio of NPB increased from 20% and 40% to 60%, the PCE values of the PSCs first increased to 13.16% and 15.04%, then decreased to 12.56%. The PSCs based on PVK doped with 40% NPB had the best performance with a V_OC_ of 0.96 V, a J_SC_ of 21.25 mA cm^−2^, and an FF of 70.03%. The resulting PCE of 15.05% is comparable with the highest PVK HTL-based PSCs, 15.8%, reported in the literature [21]. The enhancement of PCE values was caused by the simultaneous significant improvement of the V_OC_, J_SC,_ and FF values. To further verify the promotion of J_SC_, the monochromatic incident photon-to-current conversion efficiency (IPCE) spectra of the control device and device based on PVK: 40% NPB were collected, as depicted in Figure 2b. It shows a stronger spectral response in 350-620 nm after the doping of 40% NPB. The integrated J_SC_ values are 18.93 mA cm^−2^ and 20.13 mA cm^−2^ for the PVK and PVK:40% NPB-based PSC, respectively, and are in accordance with the J_SC_ values obtained from the J–V scan as shown in Figure 2a and Table 1. The reverse and forward scanned J–V curves were compared for both the control device and the device based on PVK: 40% NPB HTL to illustrate the hysteresis effect of PSCs (Figure 2c). This demonstrated that NPB doping greatly reduced the J–V hysteresis of PSCs based on PVK HTL. In addition, to demonstrate the reliability of data, the PCE value statistics from 20 devices were shown in Figure 2d, which shows narrow distributions for both the control device and 40% NPB-doped device. The errors in Table 1 were obtained by dividing the difference between the best and the average values from 20 devices by 2. 

The storage stability of unencapsulated PSCs was evaluated through aging tests, as shown in Figure 2e. After 40 days storage in an N_2_-filled glovebox, the PCE of the control PSC decayed to 73.9% of the initial value, while it was 75.1% for the 40% NPB-doped device. This result suggests that NPB doping improved the device storage stability. The light stability of unencapsulated PSCs was also examined by collecting J–V data at one-minute intervals under continuous illustration by 1 sun (100 mW cm^−2^ AM 1.5 G). The change of PCE over time is shown in Figure 2f. After 5 min illustration, the PCE values were reduced to 73% and 88% of the initial values for the control device and the PVK:40% NPB HTL device, respectively. Apparently, the light stability of PSCs based on PVK:40% NPB HTL was better.

**Figure 3:**
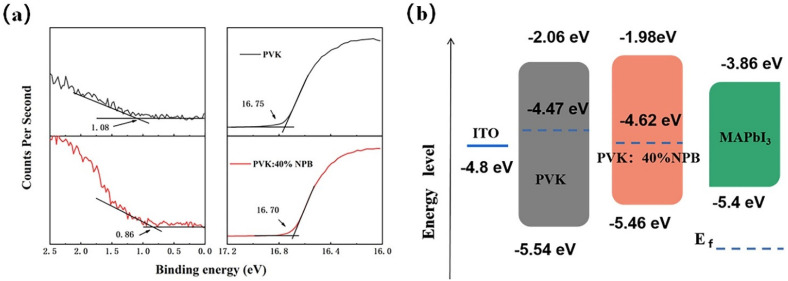
(**a**) UPS of PVK and PVK:40% NPB films and (**b**) the corresponding energy band alignment.

**Figure 4:**
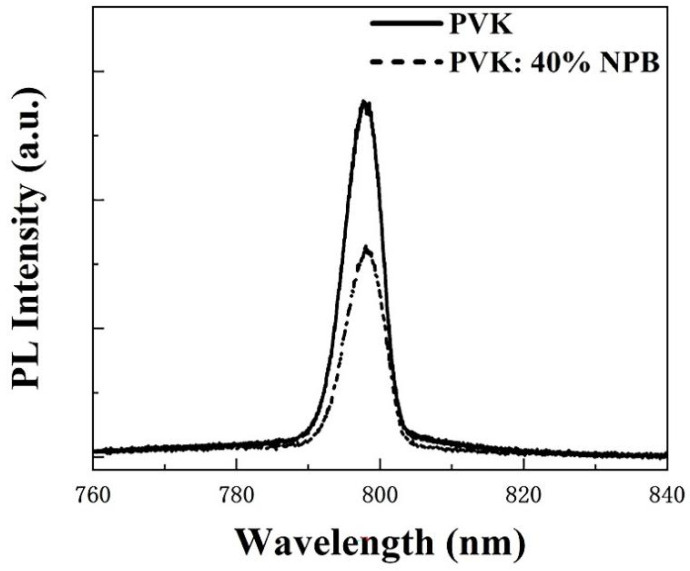
PL spectra of MAPbI_3_ on different HTLs.

Ultraviolet photoelectron spectroscopy (UPS) was conducted to explore the influence of NPB doping on the electronic energy level of HTLs, as shown in Figure 3a. According to the secondary electron cutoff (E_cutoff_) and Fermi edge (E_onset_), the calculated highest occupied molecular orbital (HOMO) values are 5.54 eV and 5.46 eV for PVK and PVK: 40% NPB, respectively, as depicted in Figure 3b. Thus, after NPB doping, the energy gap between the HOMO of HTL and MAPbI_3_ was reduced from 0.14 eV to 0.06 eV. The improvement of energy alignment between HTL and MAPbI_3_ is mainly responsible for the increase in V_OC_ after NPB doping [22]. This also suggests more efficient hole extraction efficiency, which contributed to the promotion of the J_SC_ of PSCs. The PL spectra of MAPbI_3_ on different HTLs were measured, as shown in Figure 4. The lower PL intensity of MAPbI_3_ on PVK: 40% NPB HTL indicates more efficient hole extraction efficiency [23].

**Figure 5:**
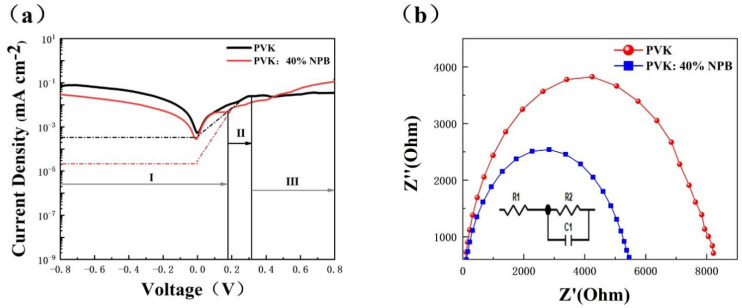
(**a**) Dark J−V curves and (**b**) Nyquist plot of PSCs.

Dark J−V data were collected to investigate the charge carrier transport dynamic of PSCs. As shown in Figure 5a, dark J–V curves can be divided into three parts [24]. Region I is a straight line of the J−V curve between negative voltage and low positive voltage, which represents the leakage current. The slope is controlled by 1/ R_sh_ (shunt resistance). Region II is an exponential line at intermediate positive voltages, indicating that the J in region II is mainly composed of the diffusion current and the recombination current [25]. Region III is a straight line at high voltage, whose slope is controlled by 1/R_s_ (series resistance). In region I, the slope of the PVK: 40% NPB device is lower than that of the control device, which is reverse in region III. This implies a larger R_sh_ and smaller R_s_ of the PVK:40% NPB device. A larger R_sh_ corresponds to a higher V_OC_, and a smaller R_s_ will result in a higher J_SC_ [26,27]. In region II, the reverse saturation current density (J_0_) can be evaluated from the intercept of the linear fitting in the exponential range, which is about 10^−3^ and 10^−5^ mA cm^−2^ for the control device and PVK: 40% NPB device, respectively. The exponent of the V_OC_ is inversely proportional to J_0_ [28]. Thus, dark J–V curves suggest that the promotion of the V_OC_ and J_SC_ of PSCs after the addition of NPB in PVK can be ascribed to the decrease in R_s_ and J_0_ and the increase in R_sh_. 

The electrochemical impedance spectroscopy (EIS) was also used to investigate the recombination inside PSCs. Figure 5b shows the Nyquist plot under dark conditions. The composite resistance (R_rec_) of the PVK:40% NPB device is 9208/Ω, which is larger than 4166/Ω of the control device. The increase in R_rec_ indicates the suppressed charge carrier recombination inside the PSC [29,30].

### 3.2. Morphology and Properties of Perovskite Films

**Figure 6:**
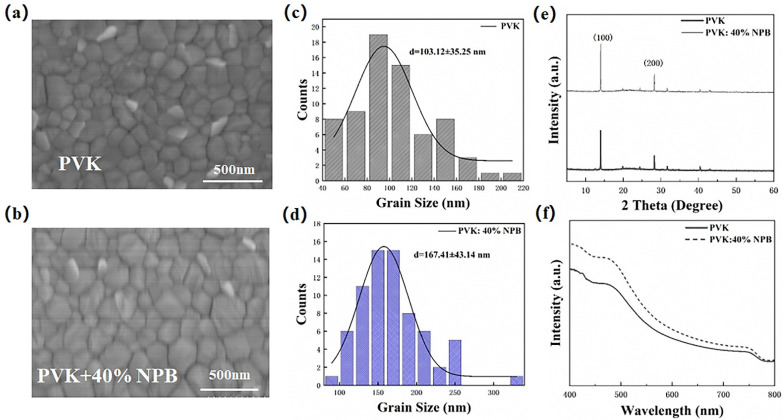
Top-view SEM images and the corresponding grain size distribution of MAPbI_3_ film on top of (**a**,**c**) PVK and (**b**,**d**) PVK: 40% NPB; (**e**) XRD patterns and (**f**) UV–vis absorption spectra of MAPbI_3_ films.

The morphology of the perovskite layer has a great influence on the performance of PSCs [31], which was then detected to explore the reason responsible for the performance enhancement of PSCs. Figure 6 shows the top-view SEM images of perovskite films on different HTLs. The corresponding grain size values were estimated by using Nano measure 1.2 and are depicted in Figure 6c,d. The average grain size of perovskite on pristine PVK HTL was 103 nm, which increased to 167 nm on PVK: 40% NPB. A larger grain size of the perovskite layer is usually caused by the increased crystallinity, which was then verified by collecting XRD patterns of perovskite films. It can be seen from Figure 6e that both patterns show characteristic peaks of orthogonal crystal structure of MAPbI_3_ at 14.1° and 28.2°, suggesting the unchanged growth direction of the MAPbI_3_ crystal, while the absolute intensities of both the (110) and (220) peaks of MAPbI_3_ on PVK: 40%NPB films are obviously higher than that of the pristine one, indicating the enhanced crystallinity of MAPbI_3_. The ultraviolet–visible (UV–Vis) absorption spectra of perovskite films deposited on different HTLs in Figure 6f show an increased absorption intensity in the range of 400–800 nm after NPB doping. This can be ascribed to perovskite crystallinity improvement [32]. The enhanced light absorption is one of the reasons responsible for the increase in J_SC_.

**Figure 7:**
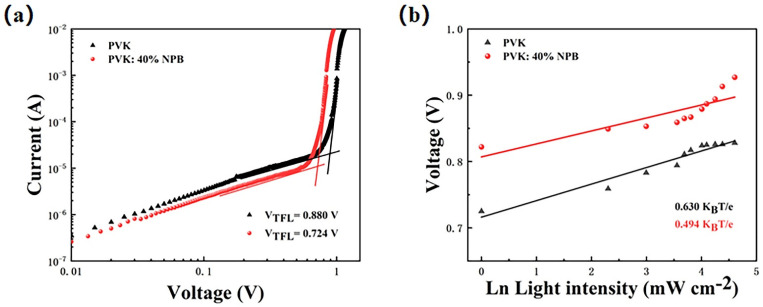
(**a**) Hole-only device based on different HTLs; (**b**) light intensity dependence of V_OC_. The light intensity increased from 1 to 100 mW cm^−2^.

The perovskite film with the large grain size contains less defect density of state, which can be estimated by using J–V curve of a single-carrier device. Thus, the hole-only device with a structure of ITO/HTL/MAPbI_3_ (260 nm)/PTAA (25 nm)/Ag (100 nm) was prepared. The density of defects can be calculated by formula (1) [33,34]:

(1) $$N_{defect}=\frac{2\varepsilon \varepsilon_{0}V_{TFL}}{qL^{2}}$$

where *ε* is the dielectric constant, *ε*_0_ is the vacuum dielectric constant, *q* is the elementary charge, and *L* is the thickness of the perovskite layer. *V_TFL_* is the voltage at the node of the J–V curve in Figure 7a, which is the trap-filling limit voltage. It can be seen from the figure that the current density increases rapidly when the bias voltage exceeds the node. This shows that the defect state is almost completely filled. The *V_TFL_* values are 0.880 V and 0.724 V for the control and PVK: 40% NPB devices, respectively. The corresponding *N_defect_* values are estimated to be 3.5 × 10^16^ and 2.9 × 10^16^ cm^−3^, respectively. The reduction of the defect density is beneficial to suppress the recombination of carriers, which is consistent with the result obtained by the EIS test. The dependence of V_OC_ on light intensity was also tested to deeply explore the carrier recombination process caused by the defect state in the optoelectronic device. Figure 7b shows the scatter plot of V_OC_ with respect to ln*(I)*, according to the following formula (2) [35]:

(2) $$\delta V_{\mathrm{OC}}=n\left(\frac{K_{B}T}{q}\right)\ln\left(I\right)+constant$$

where *n* is the ideal factor, *K_B_* is Boltzmann’s constant, *q* is the elementary charge, *T* is the absolute temperature, and *I* is the light intensity. The slope of the line after fitting in the figure is *n (K_B_T e*^−1^*)*, which is 0.630 and 0.494 *K_B_T e*^−1^ for the control and PVK:40% NPB device, respectively [36]. The decrease in the slope value means that NPB doping can effectively reduce the trap-assisted carrier recombination [37], which is in accordance with the above results.

### 3.3. Working Mechanism Analysis

**Figure 8:**
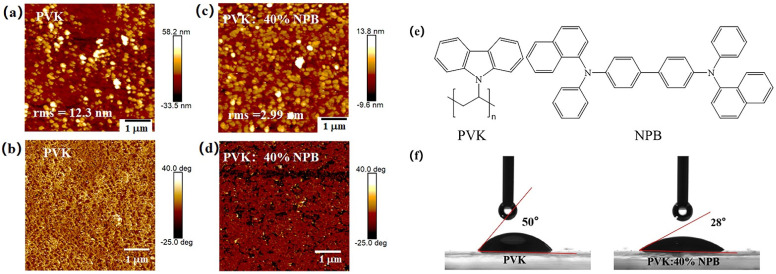
AFM images of HTLs: (**a**,**c**) height images and (**b**,**d**) phase images; (**e**) molecular structure of PVK and NPB; and (**f**) contact angles of perovskite precursor solution on HTLs.

The morphology improvement of the MAPbI_3_ layer with a larger grain size and less defect density induced the promotion of PCE of PSCs. To explore the reason responsible for the morphology improvement of MAPbI_3_, the surface topography of HTL layers was observed by using AFM, as shown in Figure 8. After adding 40% NPB in PVK, the root mean-square (rms) roughness of the HTL decreased from 12.3 nm to 2.99 nm. A smoother surface of HTL would decrease the nucleation sites for perovskite to grow and provide a better contact with the perovskite layer [38]. The C–H… π interaction between methyl groups in the PVK and the phenyl rings in the NPB interlaces with π−π stacking chains resulted in a packing motif quite similar to the conformation of polymer chains (Figure 8e). Thus, the intermolecular aggregation in the PVK was suppressed [39]. PVK chains were well dispersed within the NPB matrix due to the similar repeating unit between them, leading to a flat and smooth surface [40,41].

The contact angles of the MAPbI_3_ precursor solution on different HTL films were measured as shown in Figure 8f. For the pristine PVK substrate, the contact angle is about 50°, whereas it is 28° for the NPB-doped PVK, indicating the increased wettability. Good wettability of the perovskite precursor solution on the substrate is beneficial for the crystallization of perovskite film [42].

## 4. Discussion

With the NPB in PVK, the energy gap between the HOMO of HTL and MAPbI_3_ was reduced, leading to the enhancement of the V_OC_. The smoother surface and better hydrophilicity of the NPB-doped HTL gave rise to the morphology improvement of MAPbI_3_. MAPbI_3_ film with a larger grain size and less defect density was obtained. The reduction of R_s_ and increase in R_sh_ and UV–Vis absorption intensity are mainly responsible for the enhancement of J_SC_ and FF. Finally, a PCE promotion from 11.23% to 15.04% was achieved. This work demonstrates the great potential of PVK HTL-based PSCs.

## Figures and Tables

**Table 1:** Summary of detailed performance parameters of PSCs.

HTL	V_OC_ (V)	J_SC_ (mA cm^−2^)		FF (%)	PCE (%)
		from J–V Test	Integrated from IPCE		
PVK	0.85	20.23 ± 0.61	18.93	65.11 ± 2.32	11.23 ± 0.61
PVK:20%NPB	0.92	21.41 ± 0.65	-	66.80 ± 1.96	13.16 ± 0.59
PVK:40%NPB	0.96	21.25 ± 0.57	20.13	70.03 ± 1.81	15.04 ± 0.65
PVK:60%NPB	0.91	18.94 ± 0.73	-	73.19 ± 2.23	12.56 ± 0.94

## Data Availability

Data sharing not applicable.

## Supplementary Materials

The following supporting information can be downloaded at: https://www.mdpi.com/article/10.3390/polym14112249/s1, Figure S1: PSCs based on PVK: (a) different concentration and fixed spin-coating speed of 4000 rpm and (b) fixed concentration of 1 mg/mL and different spin-coating speed; Table S1: Summary of the detailed performance parameters of PSCs based on PVK with different concentration or spin-coating speed.
