# Supplementary material for: Highly Efficient Perovskite Solar Cell Based on PVK Hole Transport Layer
Source: Polymers (Basel). 2022 May 31;14(11):2249. doi: 10.3390/polym14112249 (PMC9183099; doi:10.3390/polym14112249)
Supplement: Supplementary file 1 [file polymers-14-02249-s001.zip › polymers-1721567-supplementary.pdf]

polymers

Supplementary Material: Highly Efficient Perovskite-Solar-Cell Based on PVK Hole Transport Layer

Yao Xu, Qiaoli Niu, Ling Zhang, Chaochao Yuan, Yuhui Ma, Wei Hua, Wenjin Zeng, Yonggang Min, Jingsong Huang and Ruidong Xia

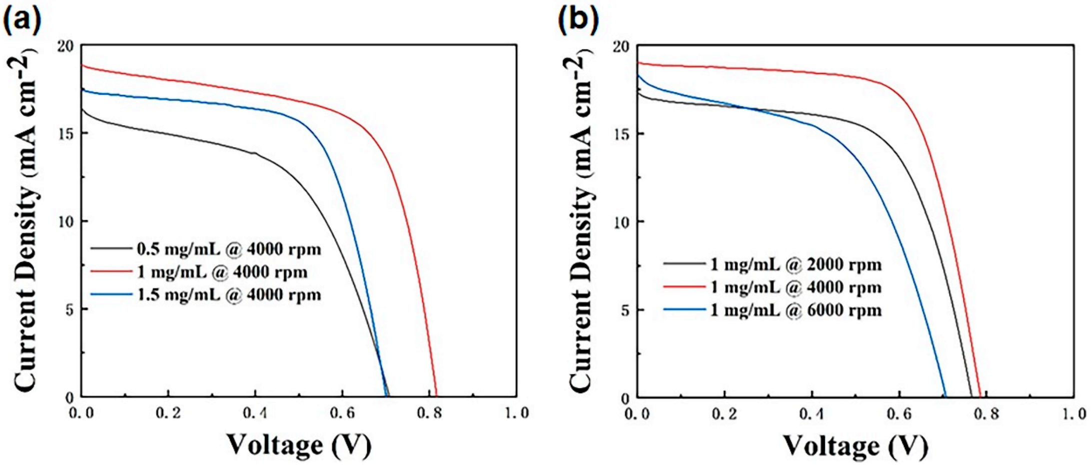

Figure S1. PSCs based on PVK: (**a**) different concentration and fixed spin-coating speed of 4000 rpm and (**b**) fixed concentration of 1 mg/mL and different spin-coating speed.

Table S1. Summary of the detailed performance parameters of PSCs based on PVK with different concentration or spin-coating speed.

Concentration of PVK (mg/mL)	Spin-coating speed (rpm)	V_{OC} (V)	J_{SC} (mA/cm^2)	FF (%)	PCE (%)
0.5	4000	0.71	16.40	53.57	6.24
1	4000	0.80	18.86	64.64	9.75
1.5	4000	0.70	17.45	65.61	8.01
1	2000	0.76	17.33	63.09	8.31
1	4000	0.78	19.04	69.16	10.27
1	6000	0.71	18.35	56.38	7.35
